# Supplementary figures and images for: Nuclear Import of Arabidopsis Poly(ADP-Ribose) Polymerase 2 Is Mediated by Importin-α and a Nuclear Localization Sequence Located Between the Predicted SAP Domains
Source: Front Plant Sci. 2018 Nov 1;9:1581. doi: 10.3389/fpls.2018.01581 (PMC6230994; doi:10.3389/fpls.2018.01581)

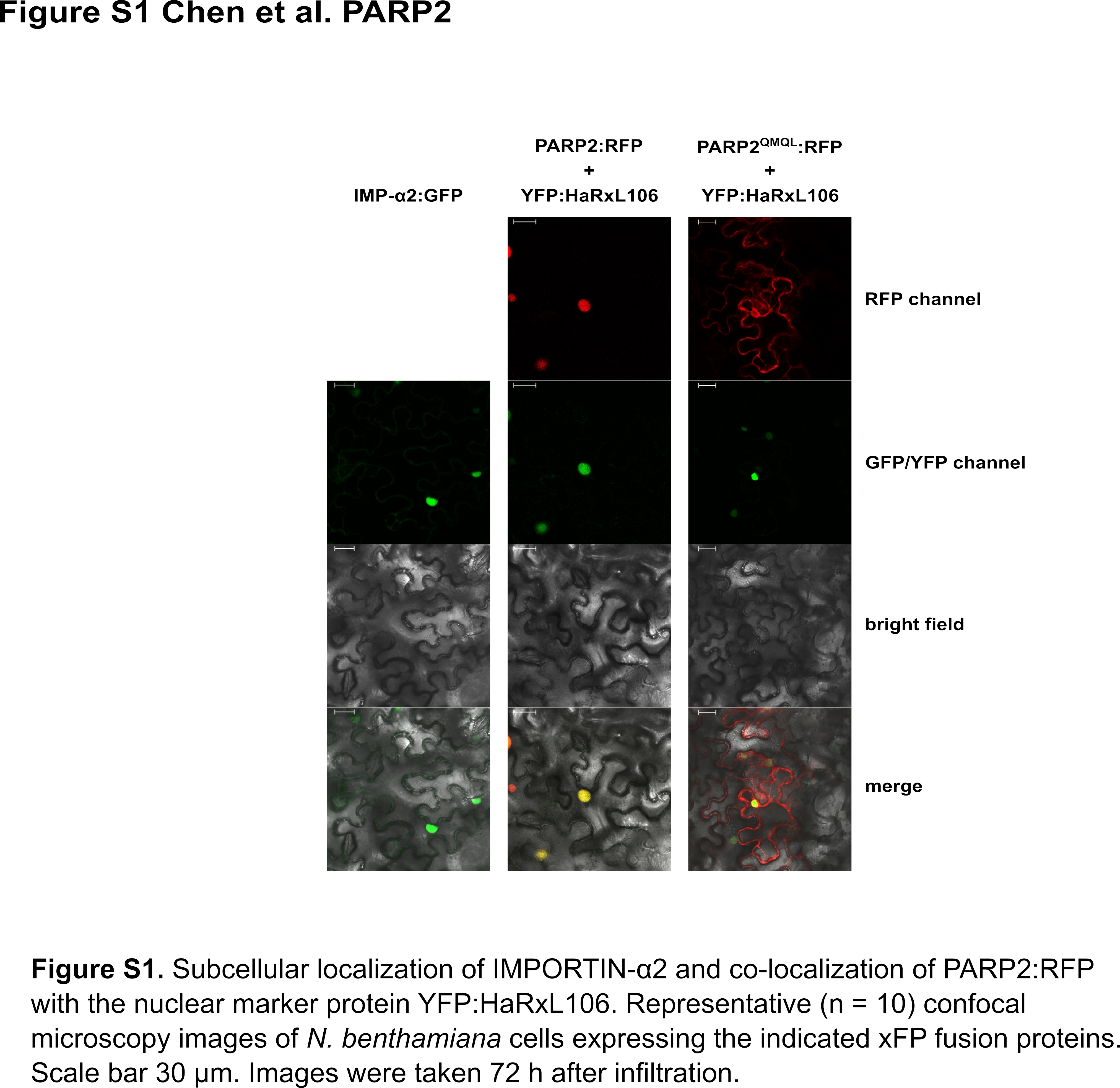

Supplement: Supplementary file 1 [file Image_1.TIF]

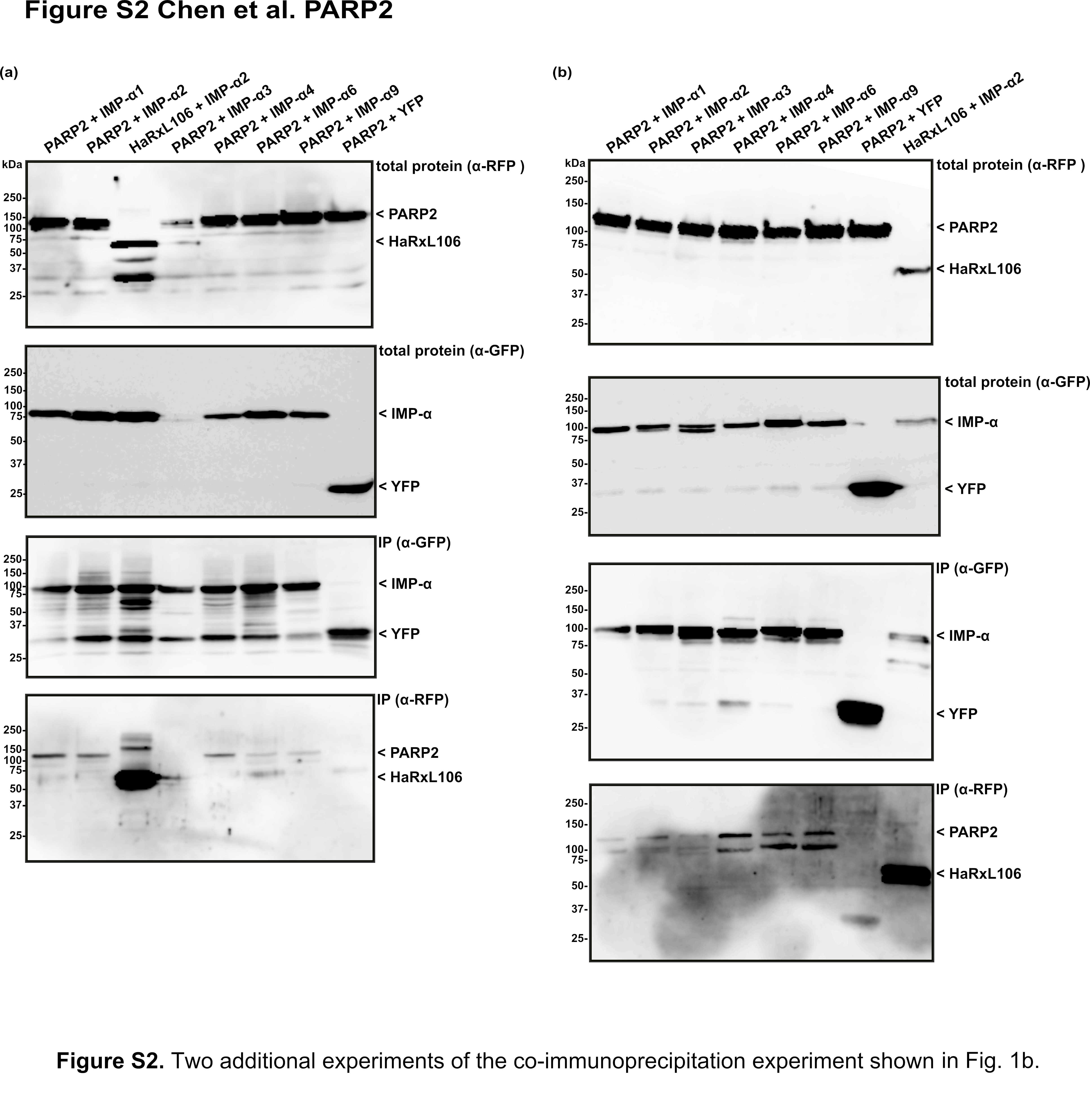

Supplement: Supplementary file 2 [file Image_2.TIF]

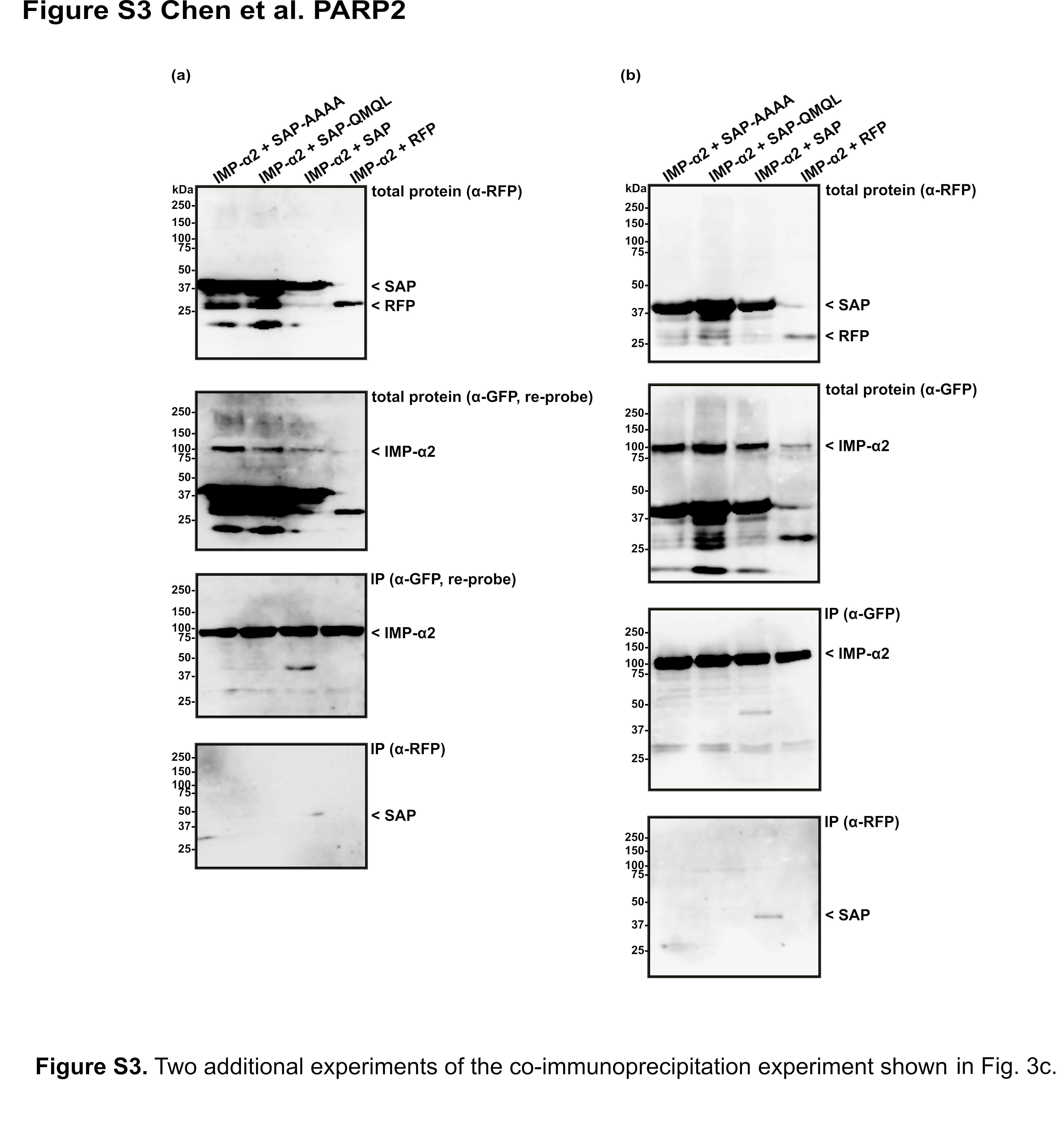

Supplement: Supplementary file 3 [file Image_3.TIF]

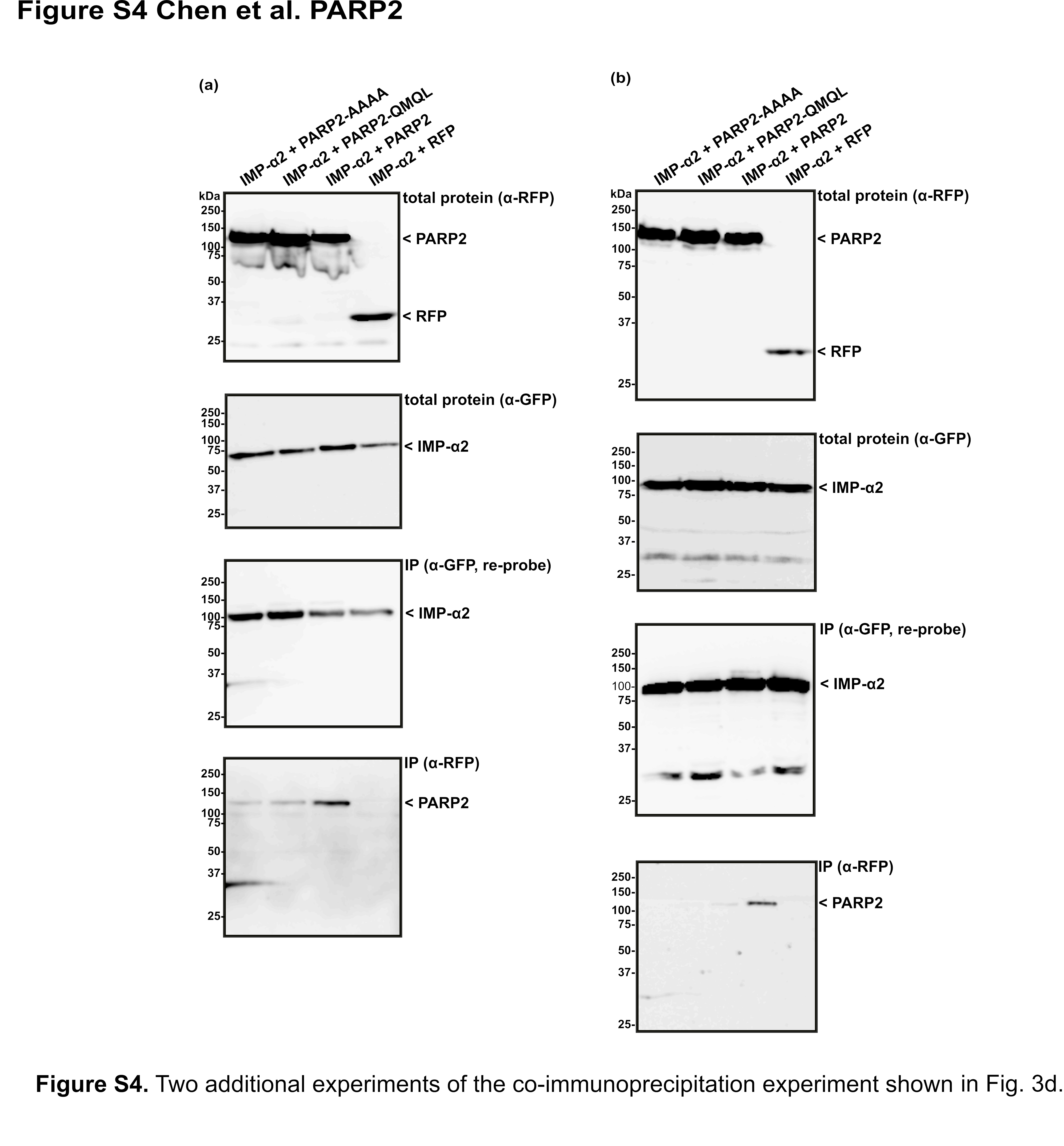

Supplement: Supplementary file 4 [file Image_4.TIF]

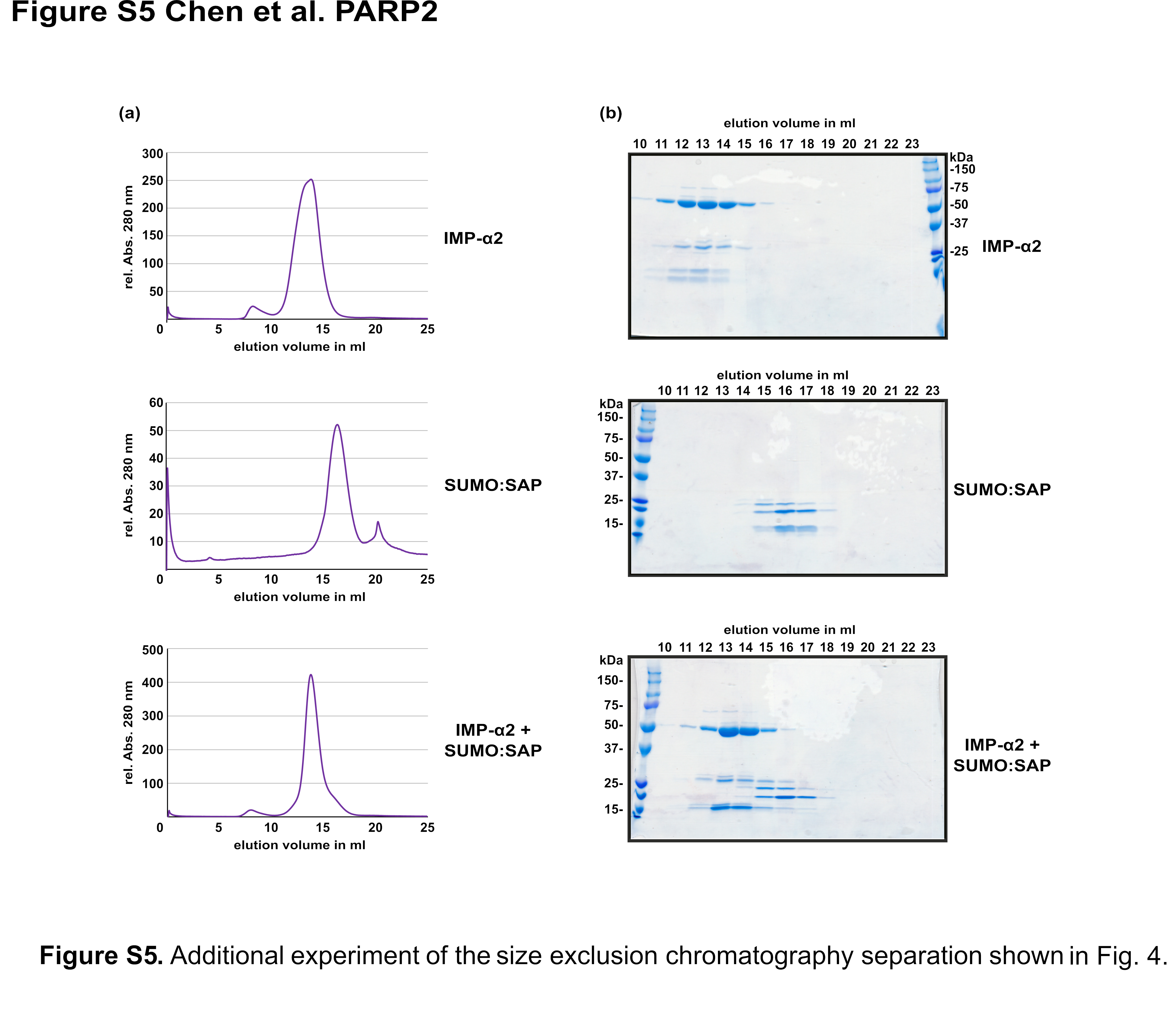

Supplement: Supplementary file 5 [file Image_5.TIF]
